# Ultrahigh anisotropic carrier mobility in ZnSb monolayers functionalized with halogen atoms†

**DOI:** 10.1039/d2ra04782a

**Published:** 2022-09-22

**Authors:** Wei Yang, Zhizi Guan, Hongfa Wang, Yongchao Chen, Hailong Wang, Junwen Li

**Affiliations:** CAS Key Laboratory of Mechanical Behavior and Design of Materials, Department of Modern Mechanics, CAS Center for Excellence in Complex System Mechanics, University of Science and Technology of China Hefei Anhui 230027 China hailwang@ustc.edu.cn; DFTWorks LLC Oakton VA 22124 USA junwen.li@dftworks.com

## Abstract

The experimental fabrication of novel two-dimensional ZnSb inspires us to explore the tunability of its fundamental physical properties. In this work, we present the density functional theory simulations on the mechanical, electronic and transport properties of the two-dimensional ZnSb monolayers functionalized with halogen atoms. It is found that the halogen atoms prefer to form ionic bonds with Sb atoms and these ZnSbX (X = Cl, Br and I) monolayers are very flexible with Young's moduli ranging from 24.02 N m^−1^ to 30.16 N m^−1^ along the armchair and zigzag directions. The pristine ZnSb monolayer sheet exhibits metallic phase while the functionalization can lead to a metal-to-semiconductor transition with band gaps as large as 0.55 eV. The transport study reveals a large tunability with the hole mobility reaching 43.44 × 10^3^ cm^2^ V^−1^ s^−1^ along the armchair direction and the electron mobility as high as 36.99 × 10^3^ cm^2^ V^−1^ s^−1^ along the zigzag direction. In contrast, the electron mobility along the armchair direction and the hole mobility along the zigzag direction are of relatively small magnitude. The ultrahigh carrier mobility together with the directional anisotropy can boost the separation of photo-excited electron–hole pairs. The finite band gaps and exceptional transport property of ZnSbX monolayers render them new materials with promising applications in flexible optoelectronic and nanoelectronic devices.

## Abstract 2

Ultrahigh carrier mobility and transport anisotropy in ZnSb monolayers functionalized with halogen atoms.

## Graphical abstract

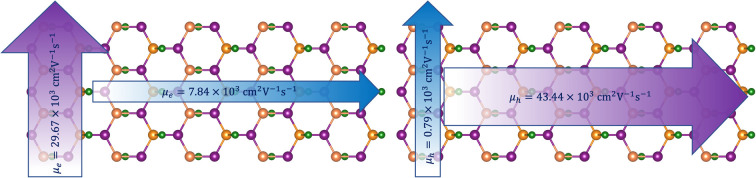

## 1 Introduction

The carrier mobility is one of the fundamental properties of semiconductors and plays an essential role in semiconductor devices because it determines the device performance in various applications such as the switching frequency in field-effect transistors, the photoconductive gain in photodetectors, and the transport properties in photovoltaic and light-emitting devices.^1^ Therefore, considerable efforts have been devoted to searching for materials with high carrier mobilities, focusing on two-dimensional (2D) systems in recent years inspired by the discovery of graphene.^2,3^ Due to the fact that the energy dispersion near Fermi level in graphene can be described by Dirac cone and the resulting massless charge carriers, the mobility in graphene can be as high as 2 × 10^5^ cm^2^ V^−1^ s^−1^ and is more than 100 times higher than that of silicon.^4^ However, the lack of a sizable gap in graphene poses limits to its applications in building field-effect transistors that require effective switching between ON/OFF states and serve as the fundamental components in semiconducting electronics. Hence, a lot of attention has been paid to investigating other two-dimensional candidate materials such as transition metal dichalcogenides, black phosphorous and MXenes.^5–8^ These two-dimensional systems are mostly exfoliated from their mother compounds with inherent two-dimensional layered configurations or synthesized *via* chemical vapor deposition method. Recently, ultra-thin zinc antimonide (ZnSb), a new member added to the two-dimensional material family, is obtained *via* lithiation transforming the three-dimensional crystal ZnSb with sp^3^ hybridization bonding to a two-dimensional structure with dominating sp^2^ bonding.^9^ The two-dimensional ZnSb monolayer sheet is found to be a metal and therefore, it would be of great significance to identify effective ways of opening up a band gap and tuning its carrier mobility for this emerging two-dimensional material to have broad applications in semiconductor electronics.^10^

The surface of two-dimensional systems plays an essential role in determining the material's properties since the large portion, if not all, of the constituent atoms are exposed on the surface. Surface functionalization has been employed efficiently tune various properties of the two-dimensional materials such as graphene, transition metal sulfides, h-BN, black phosphorus and so forth.^11–21^ For example, the fluorinated and hydrogenated graphene transform a pristine graphene from a highly conductive semimetal to an insulator and maintain the excellent high strength of graphene.^22,23^ Abellán *et al.* finds that the noncovalent organic functionalization of black phosphorus can effectively enhance the chemical stability.^17^ A pristine single-layer germanene is a quantum spin Hall insulator with a nontrivial gap of 23.4 meV while a functionalized GeI layer becomes a topological insulator with a much enhanced gap of about 0.3 eV after the passivation with iodine.^24^ Similar gap openings are also found in other two-dimensional materials such as fluorinated stanene and functionalized arsenene (AsX, X = F, OH, and CH_3_).^25–27^

In this paper, by performing density functional theory simulations, we study the functionalization effect of halogen atoms (Cl, Br and I) on the mechanical, electronic structure, and transport properties of the novel ZnSb monolayer sheet. The simulation results reveal that the functionalized ZnSb (ZnSbX, X = Cl, Br and I) monolayers behave as semiconductors. Mechanical strength analysis indicates that these ZnSbX monolayers are mechanically stable and flexible. In addition, we find the ZnSbX monolayers exhibit high hole mobility in the armchair direction and high electron mobility in the zigzag direction as well as large transport anisotropy.

This paper is organized as follows: in Section 2, we review the computational approaches employed in studying the structures, electronic structures and transport properties. In Section 3, we show the computational results and discussions. First, we study the atomic structures and the mechanical properties. Next, we look into the electronic structures, density of states and near-gap states as well as the impact of surface passivations. Lastly, we present the transport study based on deformation potential theory.

## 2 Methods

Density functional theory simulations have been carried out using Vienna *Ab initio* Simulation Package (VASP),^28–30^ with the exchange–correlation functional evaluated with the Perdew–Burke–Ernzerhof generalized gradient approximation (GGA-PBE). An energy cutoff of 500 eV is chosen for the plane wave basis set. For geometry optimization, all atomic positions are allowed to move until the force on each atom is less than 10^−3^ eV Å^−1^ with the Brillouin zone sampled by a Monkhorst–Pack special *k*-point mesh of 6 × 10 × 1. This choice of the ratio between directions is based on the consideration of the lattice constant ratio *b*/*a* = 0.61 ≈ 6/10. Moreover, to simulate the two-dimensional sheet structure and remove the spurious interactions between artificial periodic images, a vacuum region of 18 Å is used along the *z* direction normal to the sheet plane.

The carrier mobility is used to characterize the charge transport property and to evaluate the electrical performance of these materials.^31–33^ The carrier mobility is determined using the deformation potential (DP) theory derived by Bardeen and Shockley under the effective mass approximation and the electron-acoustic phonon scattering mechanism:^34,35^1
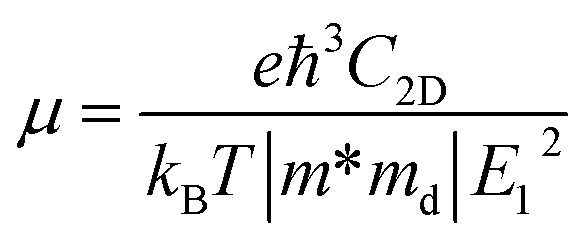
where *e* is the electron charge, ℏ is the reduced Planck constant, *C*_2D_ is the elastic modulus, *k*_B_ is the Boltzmann constant, *T* is the temperature (300 K), m* is the carrier effective mass along the transport direction with 
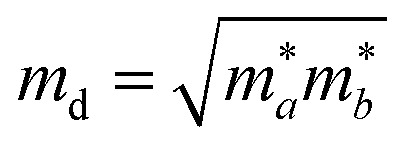
 as the carrier average effective mass, and *E*_1_ is the deformation potential constant.

The halogen atom absorption energy Δ*E*_a_ is used to describe the energetics of surface passivation, which is defined as
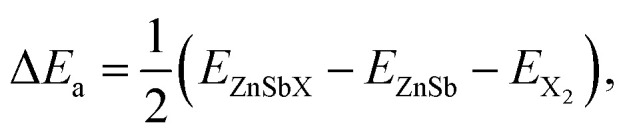
where *E*_ZnSbX_ and *E*_ZnSb_ are the total energies for the 2D ZnSbX and ZnSb unit cells, respectively, and *E*_X_2__ is the energy of a gas phase halogen molecule.

## 3 Results and discussion

### 3.1 Structural and mechanical properties

**Fig. 1:**
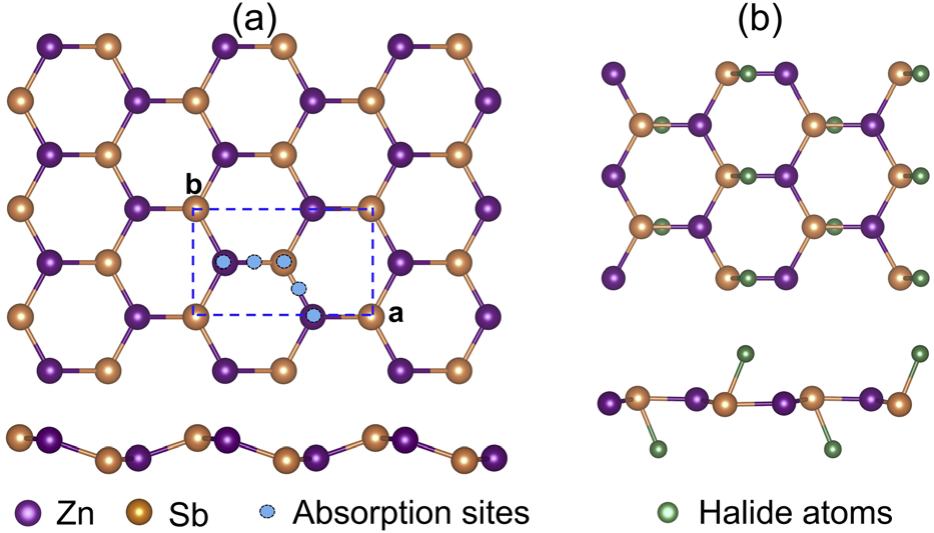
(a) The possible adsorption sites in ZnSb monolayer and (b) optimized structures with adsorption of halide atoms. The adsorption sites are represented by blue spheres. Zn, Sb and absorbed halide atoms are represented by purple, orange and cyan spheres, respectively. Here, *a* and *b* are lattice constants.

We first investigate the structural properties of the ZnSb monolayer. In Fig. 1(a) we depict the schematic of the pristine ZnSb monolayer sheet with the unit cell highlighted with dashed lines. The slightly buckled honeycomb-like crystal ZnSb consists of two Zn and two Sb atoms in the unit cell with lattice vectors *a* and *b* corresponding to armchair and zigzag directions, respectively. The lattice constants of the ZnSb monolayer are computed to be *a* = 7.45 Å and *b* = 4.52 Å, respectively. There are two inequivalent Zn–Sb bond lengths that are calculated to be 2.57 Å and 2.59 Å, comparable to the Zn–Sb bond lengths in the multilayer ZnSb system.^36^

To study how the halide atoms such as Cl, Br and I can effectively tune the various physical properties of the ZnSb monolayers, we determine the preferred adsorption sites of halide atoms on the ZnSb monolayer sheet by performing the structural relaxation with initial halide atoms placed at several possible adsorption positions, as shown in Fig. 1(a). With different starting adsorption sites, the simulations end up with the same structure for the functionalized ZnSbX (X = Cl, Br and I) monolayer sheets as depicted in Fig. 1(b). In addition, the bond lengths of Sb–Cl, Sb–Br and Sb–I for ZnSbX are calculated as 2.47 Å, 2.64 Å and 2.84 Å, respectively. When halide atoms are adsorbed to the ZnSb monolayer, it is observed that the sheet becomes flatter and the distance between two outermost layers consisting of Zn or Sb atoms decreases significantly, which leads to enhanced and close lattice constants that are *a* = 7.86 Å, *b* = 4.65 Å for ZnSbCl, *a* = 7.86 Å, *b* = 4.63 Å for ZnSbBr, and *a* = 7.86 Å, *b* = 4.64 Å for ZnSbI, respectively. Absorption energies Δ*E*_a_ are computed to be −1.70 eV, −1.55 eV and −1.90 eV for Cl, Br and I, respectively.

**Fig. 2:**
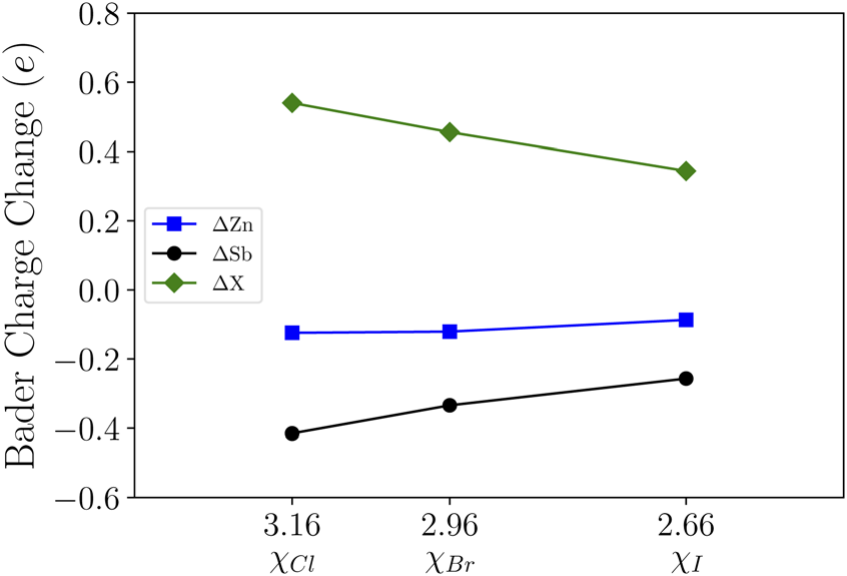
The variations in Bader charges as a function of halide atoms' electronegativities. For Zn and Sb, the Bader charges in a pristine ZnSb monolayer are taken as the reference. For X (Cl, Br, and I), the valence charge in an atomic configuration is taken as the reference.

Upon functionalization we observe the bonding charge redistribution through Bader charge analysis as presented in Table 1. Based on our calculations, the Zn atoms are positively charged while Sb atoms are negatively charged. The halide atoms of strong electronegativities with a relative order of *χ*_I_(2.66) < *χ*_Br_(2.96) < *χ*_Cl_(3.16) tend to bond with the Sb atoms, which is consistent with the structural relaxation. Taking the Bader charge of the pristine ZnSb monolayer as reference, we show the changes in Bader charges in Fig. 2. With halogen atoms adsorbed, the Zn and Sb atoms have less charges localized and the Bader charge changes follow an approximately linear pattern as a function of the electronegativity. For the halide atoms, the Bader charge change is with respect to the valence charge in an atomic configuration and the positive value indicates attraction of electrons.

**Table 1:** The Bader charges of Zn, Sb, and X ions in ZnSbX monolayers

	Zn	Sb	X
ZnSbCl	11.7722	5.2273	7.5401
ZnSbBr	11.6478	4.8121	7.4556
ZnSbI	11.6852	4.9705	7.3442

**Fig. 3:**
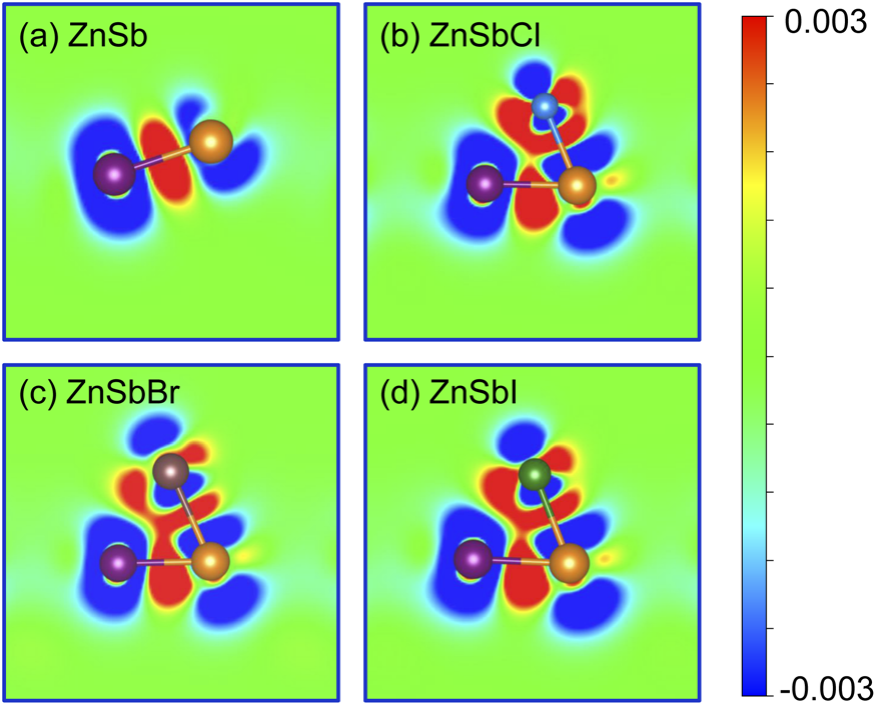
Bonding charge density for (a) ZnSb, (b) ZnSbCl, (c) ZnSbBr and (d) ZnSbI monolayer, obtained as the charge density difference between the valence charge density of the monolayer and the superposition of the valence charge density of the neutral constituent atoms. Red and blue colors indicate the electron accumulation and depletion, respectively. The color scale is in the unit of e bohr^−3^.

To further visualize the bonding state and the electronic charge redistribution of these structures, we compute the charge density difference which are obtained as the difference between the valence charge density and the superposition of the valence charge density of the constituent atoms in ZnSb and ZnSbX monolayer sheets. The computed results are plotted in Fig. 3 in which a positive value (red) indicates electron accumulation and a negative value (blue) denotes electron depletion. For the pristine ZnSb monolayer as shown in Fig. 3(a), the electron accumulation in the middle region of Zn and Sb atoms clearly shows Zn–Sb bonds prefer to combine in a covalent nature. When different halogens are adsorbed as shown in Fig. 3(b–d), there are large amount of charge transfer in the region between Sb and halogen atom, indicative of an ionic bonding.

To investigate the mechanical stability, we perform the stiffness matrix analysis for the functionalized ZnSb monolayers and in Table S1† we summarize the stiffness tensors and Young's modulus of all ZnSbX monolayers. According to our calculations, these elastic constants satisfy the Born–Huang stability criteria for rectangular structures, which are defined as:^37,38^

2 *C*_11_, *C*_66_ > 0, *C*_11_*C*_22_ > *C*_12_^2^.

Thus, all of the ZnSbX structures here are mechanically stable sheets. Based on the calculated stiffness tensors, the in-plane Young's moduli along the directions *a* and *b* are computed *via*
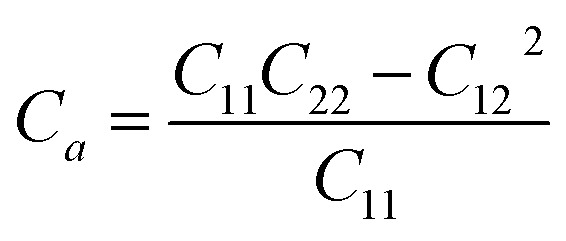
 and 
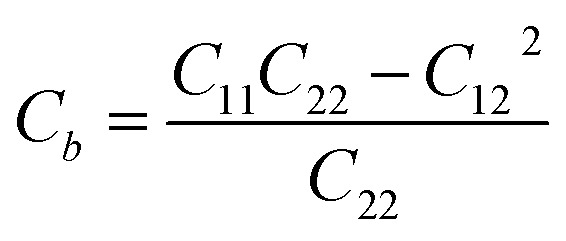
, respectively. As shown in Table 2, the in-plane Young's modulus *C*_*a*_ are generally larger than *C*_*b*_ of these materials and the maximum Young's modulus is 30.16 N m^−1^. By contrast, the stiffness of other well studied 2D materials, such as graphene, MoS_2_ and BN, whose Young's moduli are 339 N m^−1^, 122 N m^−1^ and 227 N m^−1^, respectively, are much larger than that of ZnSbX, suggesting the flexible nature of ZnSbX monolayer sheets.^39,40^

**Table 2:** Elastic constant *C*_2D_, deformation potential constant *E*_1_, charge carrier effective mass m*, and carrier mobility *μ*

	Carrier type	*C*_2D_ (N m^−1^)		*E*_1_ (eV)		m* (*m*_0_)		*μ* (×10^3^ cm^2^ V^−1^ s^−1^)	
		*a*	*b*	*a*	*b*	*a*	*b*	*a*	*b*
ZnSbCl	e	30.16	24.02	−4.88	−2.63	0.06	0.05	7.84	29.67
	h			0.40	−3.41	−0.33	−0.21	43.44	0.79
ZnSbBr	e	30.07	24.66	−5.07	−2.64	0.06	0.04	7.44	36.99
	h			0.42	−3.48	−0.36	−0.25	32.61	5.70
ZnSbI	e	30.14	25.02	−6.37	−3.43	0.06	0.04	5.72	22.37
	h			0.32	−3.77	−0.44	−0.37	34.95	0.25

### 3.2 Electronic structures

**Fig. 4:**
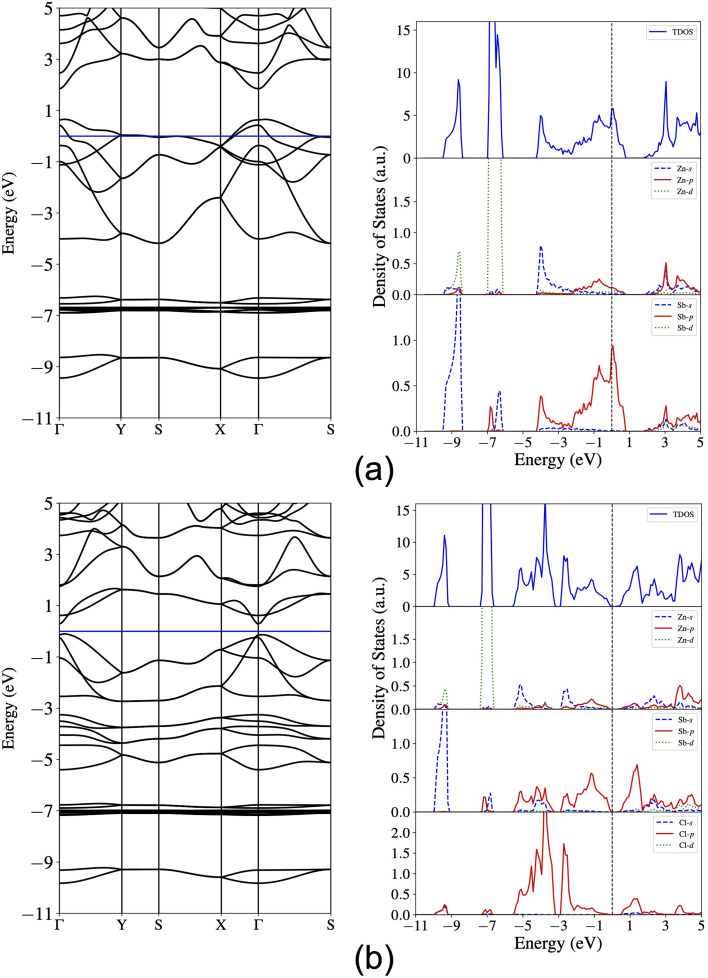
Calculated band structure and total and orbital-projected density of states for (a) ZnSb and (b) ZnSbCl monolayer. The Fermi level is set at the zero energy.

Next, we compute the electronic band structure and density of states (DOS) and present the results for the pristine monolayer in Fig. 4(a). The band crossing at the Fermi level indicates the metallicity of the pristine ZnSb sheet and the orbital-projected density of states further reveal that the energy states around the Fermi level are composed of dominantly Sb-p orbitals and marginally Zn-p orbitals. The Zn-s orbitals are mainly located in the range of −4.0 eV to −2.0 eV while Zn-d orbitals account for the flat bands lying from −7.0 eV to −6.0 eV. The Sb-s orbitals are mainly contributing to the energy states between −9.5 eV and −8.5 eV. The dominance of Sb-p orbitals around the Fermi level are consistent with the fact that halogen atoms prefer to bond with the Sb atoms and also are indicative of significant changes in electronic structures that could be induced by passivating the Sb-p orbitals.

For the ZnSbCl monolayer, we present the electronic band structure and density of states in Fig. 4(b). It exhibits a nearly direct band gap of 0.38 eV with the conduction band minimum (CBM) at the Γ point and the valence band maximum (VBM) slightly away from the Γ point. This gap value is smaller than 0.5 eV reported by Bafekry *et al.*,^41^ which could be due to the consideration of spin–orbit coupling. The orbital-projected density of states show that the conduction band states near Fermi level are mainly composed of the Sb-p and Cl-p orbitals, indicating a strong orbital interaction. The near-gap valence band states are mainly derived from the Sb-p orbitals and Cl-p orbitals dominate the valence band states at a lower energy window (−6 eV to 0 eV). Both the electronic structure and PDOS indicate the energy states from −9.5 eV to −6 eV are nearly unaffected. The flatter bands can be attributed to the enhanced lattice constants in ZnSbCl and the resulting weaker orbital interaction. The electronic structures of ZnSbBr and ZnSbI monolayers with band gaps of 0.44 eV and 0.55 eV are similar to that of ZnSbCl monolayer and are presented in Fig. S1 and S2,† respectively. The relative band gap size could be understood by looking into the energy levels of VBM and CBM of these compounds. Fig. S3† shows the partial charge density iso-surfaces of VBM and CBM and indicates that the CBM is of anti-bonding character and is mainly contributed by s orbitals of Zn and s and p orbitals of Sb while the VBM is a bonding state formed between p orbitals of Sb and Zn. Because of the localized nature of CBM, the CBM energy level could experience larger interaction with the negatively charged halogen ions. As show in Fig. 6, when changing from Cl to Br (I) for the surface functionalization, the VBM increases by 0.11 (0.25) eV while the CBM increases by 0.17 (0.42) eV, leading to a gap increase by 0.06 (0.17) eV. The effect of different halogen atom could be attributed to their different ionic radii that are 1.81 Å, 1.96 Å and 2.20 Å for Cl, Br, and I, respectively.

### 3.3 Transport property

**Fig. 5:**
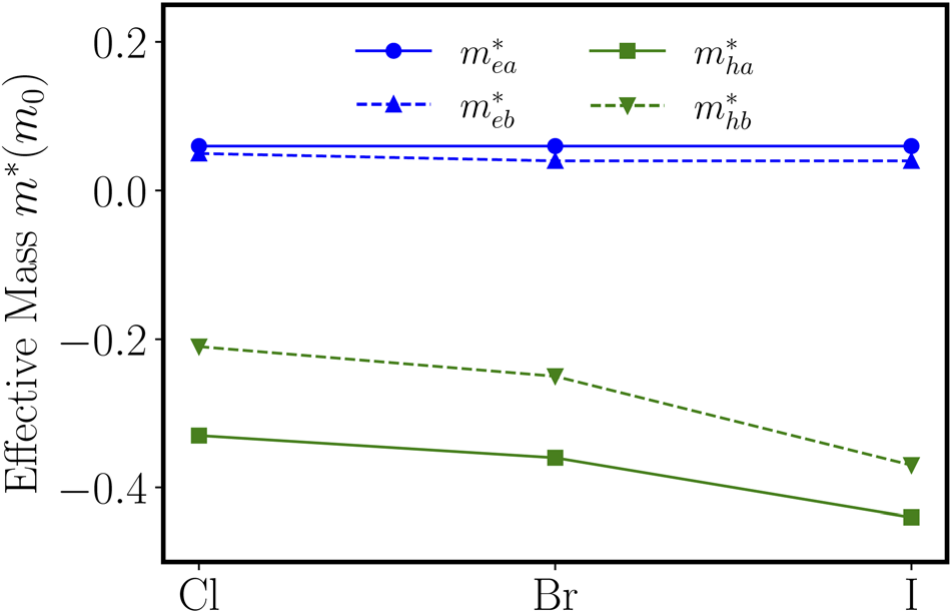
The electron effective masses 
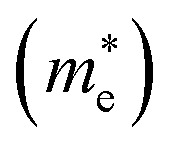
 and hole effective masses 
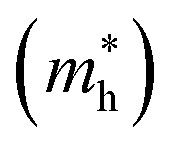
 along the *a*-direction (armchair) and *b*-direction (zigzag) for ZnSbX, respectively. Blue and green colors indicate electron and hole effective masses, respectively.

As shown in the band structures, different adsorbed atoms induce the change of band curvature, which is directly related to the effective mass of carrier (m*)^42,43^ defined as3
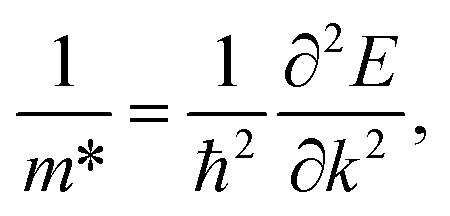
where ℏ is the reduced Planck's constant, *E* is the energy, and *k* is the momentum. In Fig. 5 we present the computed effective mass values of the electrons and holes along the *a* and *b* directions (corresponding to the directions from Γ to X point and Γ to Y point in the Brillouin zone) for ZnSbX, respectively. Due to the 2D nature and inherent anisotropy of ZnSbX monolayers, the effective masses of charge carriers also exhibit high anisotropy. In general, the effective masses of electrons and holes are relatively small in the *b* direction, and also different ZnSbX compounds will lead to different effective masses. For ZnSbCl, the effective masses of the electron 
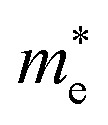
 are calculated to be 0.06*m*_e_ and 0.05*m*_e_ in the armchair and zigzag directions, respectively. The effective masses of the hole 
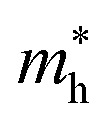
 are predicted to be 0.33*m*_e_ and 0.21*m*_e_ in the armchair and zigzag directions, respectively. For ZnSbBr and ZnSbI we observe the same relative magnitudes between the effective masses of electrons and holes as shown in Table 2.

**Fig. 6:**
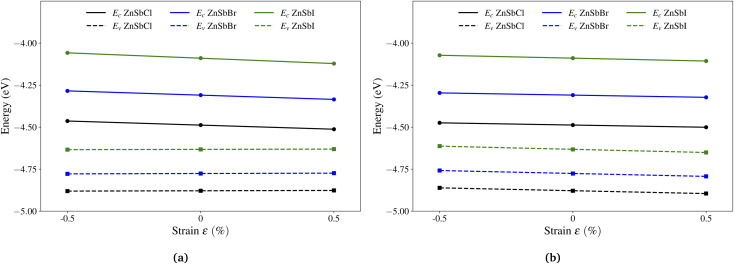
The band edge positions of conduction band minimum and valence band maximum as a function of uniaxial strain along (a) the armchair and (b) the zigzag directions, respectively. Black, blue and green lines represent the linear fitting for ZnSbCl, ZnSbBr and ZnSbI, respectively. The band energy values are computed with respect to the *x*–*y* plane averaged electrostatic potential energy in the vacuum region.

The effective mass is a crude estimation for the transport property. The carrier mobility is also dependent on the phonon scattering here evaluated through the deformation potential constant. The deformation potential constant *E*_1_ is defined as the gradient of band edge (VBM and CBM) change with respect to strain *via* Δ*E* = *E*_1_(Δ*l*/*l*_0_), in which Δ*E* is the energy shift of the band edge position with respect to the uniaxial strain Δ*l*/*l*_0_ along the orientation *a* or *b*. In Fig. 6, we plot the CBM and VBM variations as a function of the applied strains of −0.5%, 0.0% and 0.5%. To compare the VBM and CBM values of configurations under different strains, a common reference energy is needed, for which, we compute the *x*–*y* plane averaged electrostatic potential energy. The zero energy is set to the electrostatic potential energy at the vacuum region, located farthest to the monolayer sheet plane. As shown in Fig. 6(a), under uniaxial strains along direction *a*, the conduction band edge of ZnSbCl decreases much faster than the valence band edge, indicating a larger deformation potential constant for CBM. A linear fitting gives us the DP constants: *E*_1c_ = −4.88 eV and *E*_1v_ = 0.40 eV for ZnSbCl. The similar trend applies for ZnSbBr and ZbSbI whose deformation potential constants are computed to be *E*_1c_ = −5.07 eV and *E*_1v_ = 0.42 eV for ZnSbBr, and *E*_1c_ = −6.37 eV and *E*_1v_ = 0.32 eV for ZnSbI, respectively. However, along direction *b*, the situation is much different and the changes in conduction and valence band edges are very similar as demonstrated in Fig. 6(b). Linear fitting gives us the DP constants: *E*_1c_ = −2.63 eV and *E*_1v_ = −3.41 eV for ZnSbCl, *E*_1c_ = −2.64 eV and *E*_1v_ = −3.48 eV for ZnSbBr, and *E*_1c_ = −3.43 eV and *E*_1v_ = −3.77 eV for ZnSbI.

**Fig. 7:**
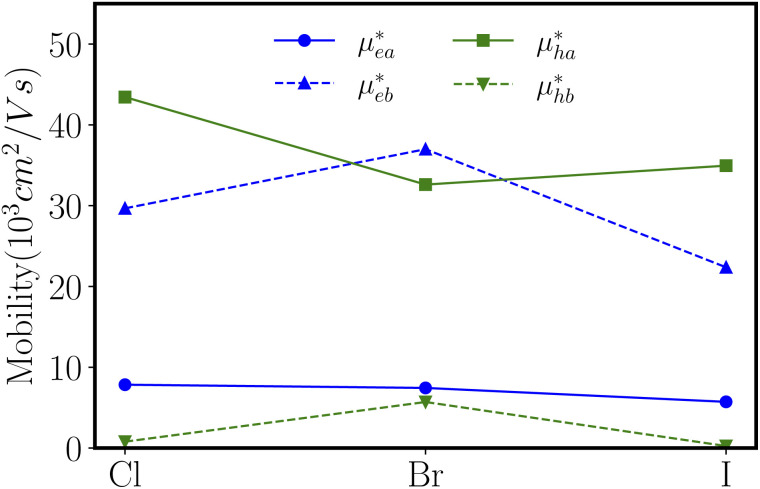
The electron mobility (*μ*_e_) and hole mobility (*μ*_h_) along the *a*-direction (armchair) and *b*-direction (zigzag) for ZnSbX, respectively. Blue and green colors indicate electron and hole mobilities, respectively.

With elastic modules, effective masses and deformation potential constants computed, we compute using eqn (1) and present the charge carrier mobilities in Fig. 7 as well as in Table 2. We first focus on the result of ZnSbCl: the electron mobilities are computed to be 7.84 × 10^3^ cm^2^ V^−1^ s^−1^ and 29.67 × 10^3^ cm^2^ V^−1^ s^−1^ in the armchair and zigzag directions, respectively; the hole mobilities are predicted to be 43.44 × 10^3^ cm^2^ V^−1^ s^−1^ and 0.79 × 10^3^ cm^2^ V^−1^ s^−1^ in the armchair and zigzag directions, respectively. The high hole mobility along the armchair direction is due to the small deformation potential constant which corresponds to a nearly flat curve as shown in Fig. 6(a). Since the deformation potential constant takes a quadratic term in the denominator of eqn (1), it has a stronger effect than other ingredients on the carrier mobility. However, the large electron mobility along the zigzag direction is a combination of relatively smaller elastic modulus and deformation potential constant given that the electron effective masses along the armchair and zigzag directions are very close. The similar relative trend is also observed for ZnSbBr and ZnSbI. The large and directionally anisotropic carrier mobility could promote the separation of electron–hole pairs and reduce the recombination rate of electrons and holes which is critical to the performance of optoelectronics devices. We can compare the carrier mobilities of ZnSbX with other two-dimensional materials with high carrier mobilities. Graphene is well known for its ultrahigh carrier mobility,^3,44,45^ which even can exceed 2 × 10^5^ cm^2^ V^−1^ s^−1^. The monolayer phosphorene has high carrier mobilities in the range of 10–26 × 10^3^ cm^2^ V^−1^ s^−1^ and also the carrier mobilities ranging from 1.92 × 10^3^ cm^2^ V^−1^ s^−1^ to 19.93 × 10^3^ cm^2^ V^−1^ s^−1^ are found in the phosphorous derivatives such as two-dimensional hittorfene, GeP_3_, InP_3_, CaP_3_, and P_3_S.^46–51^ Moreover, for two-dimensional materials, the carrier mobility could be enhanced by making rippled structures. As demonstrated in MoS_2_, the introduction of ripples could lead to reduced electron–phonon scattering and therefore, enhanced room temperature mobility reaching 900 cm^2^ V^−1^ s^−1^, much larger than the mobility of 200–410 cm^2^ V^−1^ s^−1^ predicted in flat MoS_2_.^52^

It is noted that the ultrahigh carrier mobility predicted herein is based on the simplified approach taking into account acoustic phonon scattering at room temperature. At considerably high temperature and large carrier densities, other limiting factors such as optical phonon scattering and electron–electron scattering would become more important. Hence, more sophisticated simulations are needed to obtain more accurate quantitative estimation of the charge carrier mobilities.

## 4 Summary

Using density functional theory simulations, we have investigated the effect of functionalization on the physical properties of the zinc antimonide monolayer. By halogen atom functionalization, the modified ZnSb monolayers are transformed into semiconductors with sizable band gaps up to 0.55 eV. The mechanical property analysis indicates that ZnSbX monolayers are flexible materials with Young's moduli ranging from 24.02 N m^−1^ to 30.16 N m^−1^ much smaller than other well studied two-dimensional materials such as graphene, MoS_2_ and BN. Meanwhile, these ZnSbX monolayers prefer to have an electron transport along the zigzag direction with the electron mobility reaching 36.99 × 10^3^ cm^2^ V^−1^ s^−1^ and a hole transport along the armchair direction with the hole mobility as high as 43.44 × 10^3^ cm^2^ V^−1^ s^−1^. The transport anisotropy and high carrier mobility are essential to ensure optimal performance such as a high switching frequency and low power dissipation in the nanoelectronic devices and reduced recombination rate of electron–hole pairs. Therefore, the simulations suggest that halogen functionalization effectively modulates the physical properties of ZnSbX monolayers that could find applications in novel electronics and optoelectronics.

## Conflicts of interest

There are no conflicts to declare.

## Supplementary Material

RA-012-D2RA04782A-s001
