# Supplementary material for: Ultrahigh anisotropic carrier mobility in ZnSb monolayers functionalized with halogen atoms
Source: RSC Adv. 2022 Sep 22;12(41):26994–7001. doi: 10.1039/d2ra04782a (PMC9493468; doi:10.1039/d2ra04782a)
Supplement: RA-012-D2RA04782A-s001 [file RA-012-D2RA04782A-s001.pdf]

Electronic Supplementary Material (ESI) for RSC Advances.
This journal is © The Royal Society of Chemistry 2022

Supporting information

Ultrahigh anisotropic carrier mobility in ZnSb monolayers functionalized with halogen atoms

Zhizi Guan[a], Wei Yang[a], Hongfa Wang[a], Yongchao Chen[a], Hailong Wang[a], and Junwen Li[b]

[a] CAS Key Laboratory of Mechanical Behavior and Design of Materials, Department of Modern Mechanics, CAS Center for Excellence in Complex System Mechanics, University of Science and Technology of China, Hefei, Anhui 230027, China
E-mail: hailwang@ustc.edu.cn

[b] DFTWorks LLC, Oakton, VA 22124, USA
E-mail: junwen.li@dftworks.com

To check the mechanical stability of functionalized ZnSb monolayers, we carry out the calculations of elastic stiffness constants of ZnSbX (X=Cl, Br, I) monolayers. With the Born-Huang stability criteria as described by

$$C_{11} > 0,\ C_{22} > 0,\ C_{11} > |C_{12}|,\ C_{66} > 0,$$

the 2D ZnSbX monolayers are found to be mechanically stable as shown in table S1.

Table S1. Elastic Stiffness Constants of functionalized 2D ZnSbX monolayer.

	C_{11}(N m^{-1})	C_{12}(N m^{-1})	C_{22}(N m^{-1})	C_{66}(N m^{-1})
ZnSbCl	33.54	9.51	26.72	1.82
ZnSbBr	34.61	11.34	28.38	1.80
ZnSbI	36.09	13.35	29.96	1.87

According to Eq. 1, the mobility is inversely proportional to the temperature. Taking the mobility computed at 300 K as a reference μ_0, the mobility at other temperatures can be obtained via $\mu(\mathrm{T}) = \mu_0 \times 300/\mathrm{T}$. In Tab. S2, the mobilities at 200 K and 400 K are listed and the values at 300 K are also presented for comparison.

Table S2. Carrier mobilities at temperatures T = 200 K, 300 K and 400 K.

		T = 200 K		T = 300 K		T = 400 K	
		$\mu(\times 10^3 \mathrm{cm^2V^{-1}s^{-1}})$		$\mu(\times 10^3 \mathrm{cm^2V^{-1}s^{-1}})$		$\mu(\times 10^3 \mathrm{cm^2V^{-1}s^{-1}})$	
		a	*b*	*a*	*b*	*a*	*b*
ZnSbCl	*e*	11.76	44.51	7.84	29.67	5.88	22.25
	h	65.16	1.19	43.44	0.79	32.58	0.59
ZnSbBr	*e*	11.16	55.49	7.44	36.99	5.58	27.74
	h	47.42	8.55	32.61	5.70	23.71	4.28
ZnSbI	*e*	8.58	33.56	5.72	22.37	4.29	16.78
	h	52.43	0.36	34.95	0.25	26.21	0.19

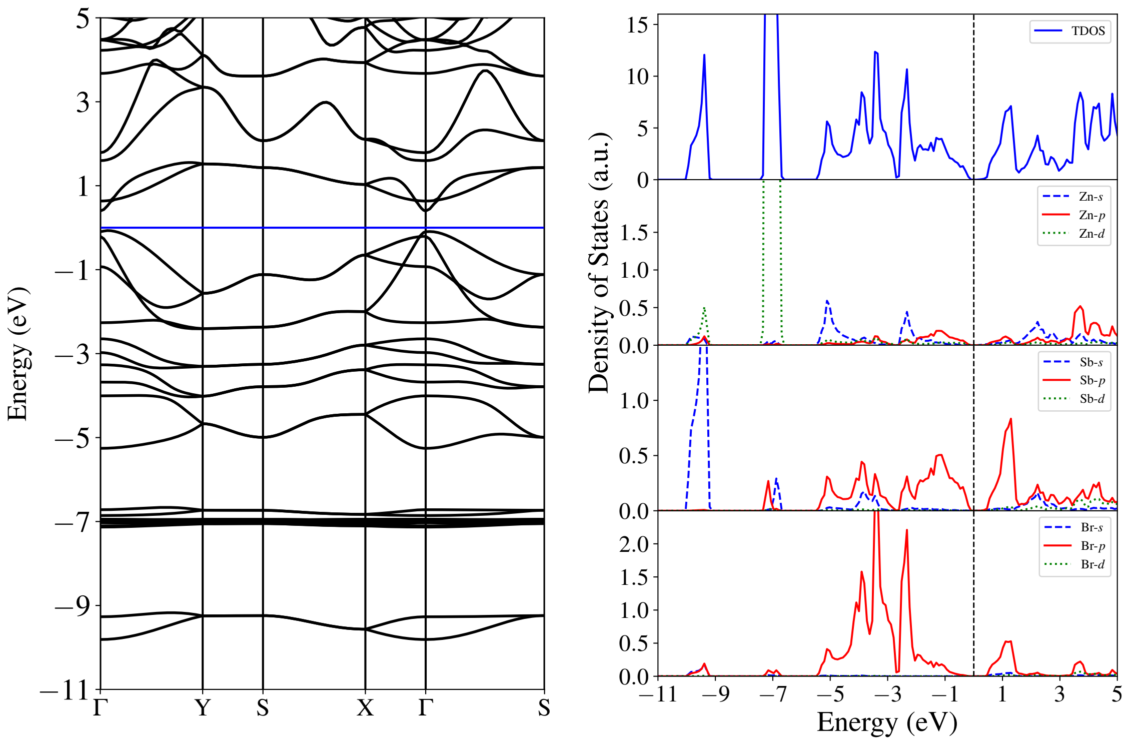

(a) (b)

Figure S1. (a) Electronic band structure and (b) total and orbital-projected density of states for the ZnSbBr monolayer sheet. The Fermi level is set to zero.

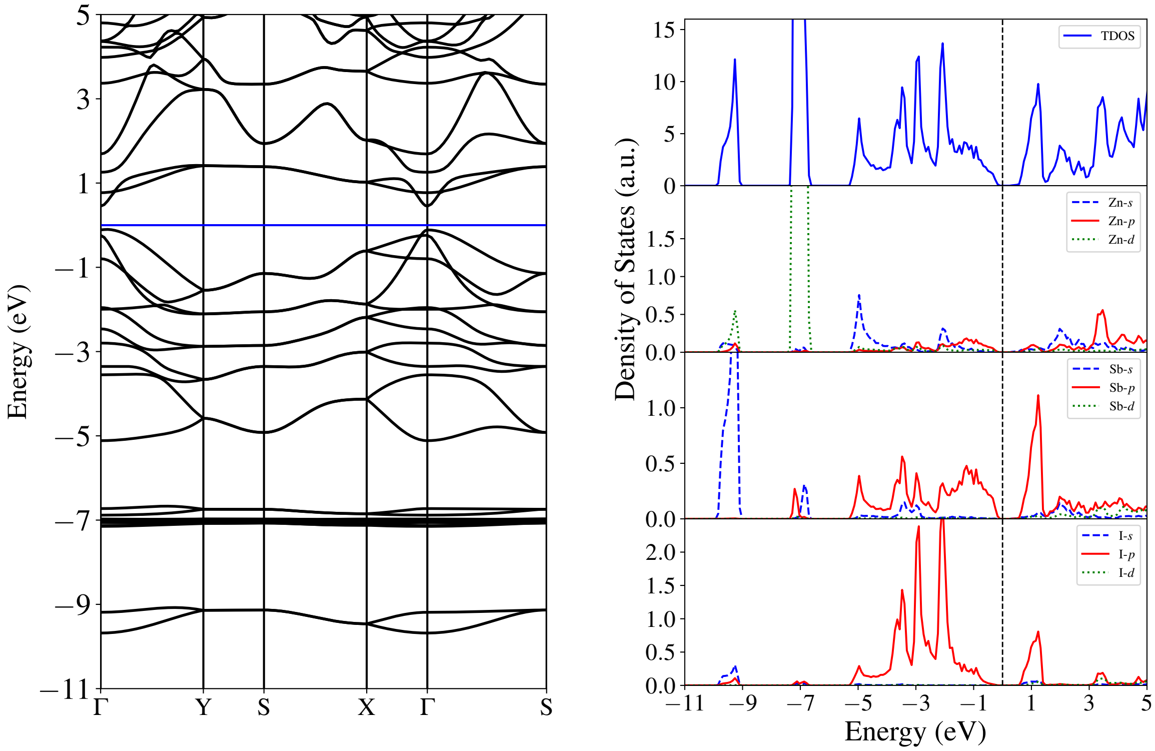

(a) (b)

Figure S2. (a) Electronic band structure and (b) total and orbital-projected density of states for the ZnSbI monolayer sheet. The Fermi level is set to zero.

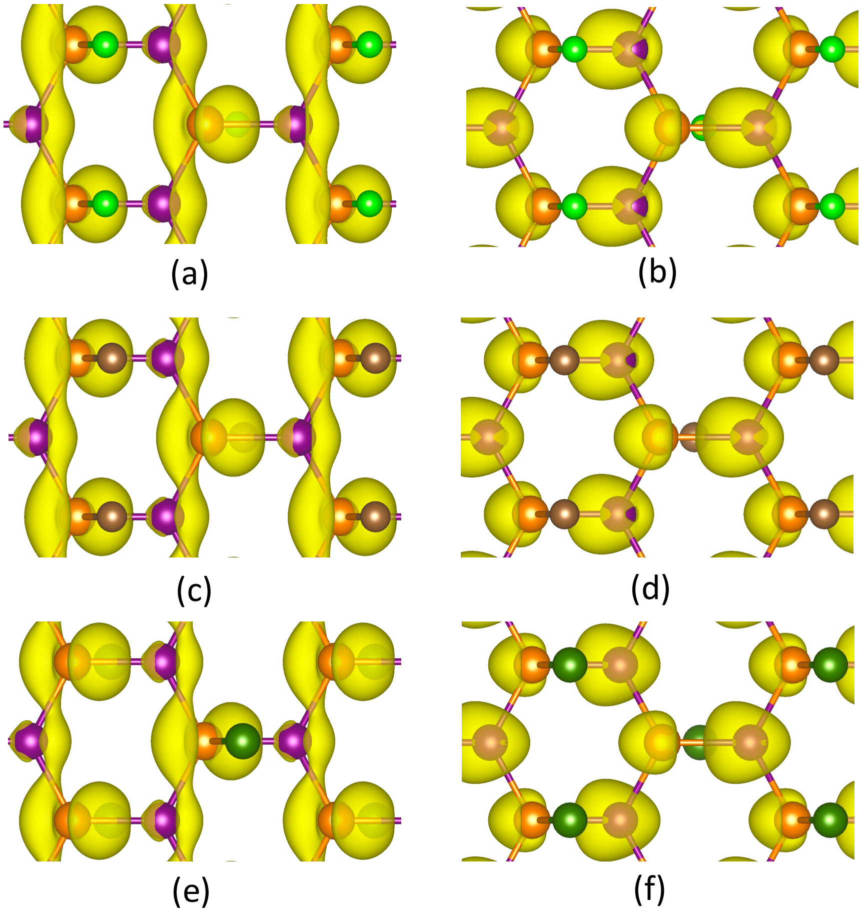

Figure S3. Partial charge density iso-surfaces (a) and (b) correspond to the valence band maximum and conduction band minimum at Γ point of ZnSbCl, respectively. Partial charge density iso-surfaces (c) and (d) correspond to the valence band maximum and conduction band minimum at Γ point of ZnSbBr, respectively. Partial charge density iso-surfaces (e) and (f) correspond to the valence band maximum and conduction band minimum at Γ point of ZnSbI, respectively.
